# Teprotumumab for Treatment of Pretibial Myxedema

**DOI:** 10.1210/jcemcr/luac037

**Published:** 2023-01-23

**Authors:** Michelangelo P Reyes, John Cabrera, Jasvir Singh, Dennis Turnbull

**Affiliations:** Sierra View Medical Center, Porterville, California 93257, USA; Sierra View Medical Center, Porterville, California 93257, USA; Sierra View Medical Center, Porterville, California 93257, USA; Sierra View Medical Center, Porterville, California 93257, USA

**Keywords:** teprotumumab, pretibial myxedema, thyroid eye disease

## Abstract

Pretibial myxedema (PTM), also called thyroid dermopathy, is a dreaded and potentially debilitating manifestation of thyroid disease, more commonly Graves' disease, which can occur at any time over the course of the disease. No substantial long-term therapies have been able to target the condition, and management has typically been supportive (eg, compression socks, weight loss), with courses of moderate-intensity steroids. Teprotumumab has been approved for the management of thyroid eye disease (TED), and it is believed that the 2 share a similar pathophysiology likely related to type 1 insulin-like growth factor receptor, which may explain why some patients have also experienced improvement in PTM. Here we present a patient who received 8 doses of teprotumumab for TED who, over the course of management and into follow-up, experienced significant improvement in her pretibial myxedema. The patient noted considerable improvement in quality of life and ability to perform daily activities. We present this case to consider further investigation into the utilization of teprotumumab for thyroid disease–related PTM in patients with impaired quality of life.

## Introduction

Pretibial myxedema (PTM) is a commonly associated manifestation of Graves' disease, as it occurs in 0.5% to 4.3% of patients (1). A skin lesion that presents as a nonpitting edema on the anterior and/or lateral surfaces of the legs bilaterally, it can present with demarcated papules/nodules and have an “orange-peel” appearance. Immunohistological studies show that myxomatous materials in cutis from a patient with PTM with Graves' disease were found to be mainly hyaluronic acid, which had accumulated extensively in the upper dermis, likely originating from deposition by fibroblasts in the lower layers of the dermis. The lesions come from the deposition of glycosaminoglycans/hyaluronic acid in the connective tissue; the cells contain thyrotropin receptors that are stimulated by the circulating thyrotropin-receptor antibodies. A similar pathophysiology occurs in thyroid eye disease (TED). The diagnosis of PTM is usually made clinically; biopsy is normally not indicated in the setting of a patient with active Graves' disease/hyperthyroidism. Historically, treatment for PTM has been limited. Typically, if patients were asymptomatic no treatment was provided, and for symptomatic patients, risk factor modifications are undertaken (weight loss/compression stockings), and moderate-potency intralesional/topical or oral corticosteroids prescribed. But despite these treatment options, patients often find no resolution of symptoms. However, teprotumumab has recently become a potential treatment option for patients with severe PTM (2-4). It is a monoclonal antibody inhibitor of type 1 insulin-like growth factor receptor (IGF-1R). It has already been approved for the treatment of TED. There have been 3 case reports that have shown teprotumumab to be successful in the resolution of PTM (2-4). The data are limited to long-term outcomes, and research is ongoing.

## Case Presentation

The patient is a 71-year-old White woman with a history of Graves' disease in remission after radioactive iodine now with postprocedural hypothyroidism who presented to the endocrinology clinic. The patient was referred to the clinic by her primary care provider. The patient also presented with associated TED and severe bilateral myxedema of the lower extremities.

## Diagnostic Assessment

**Figure 1.**
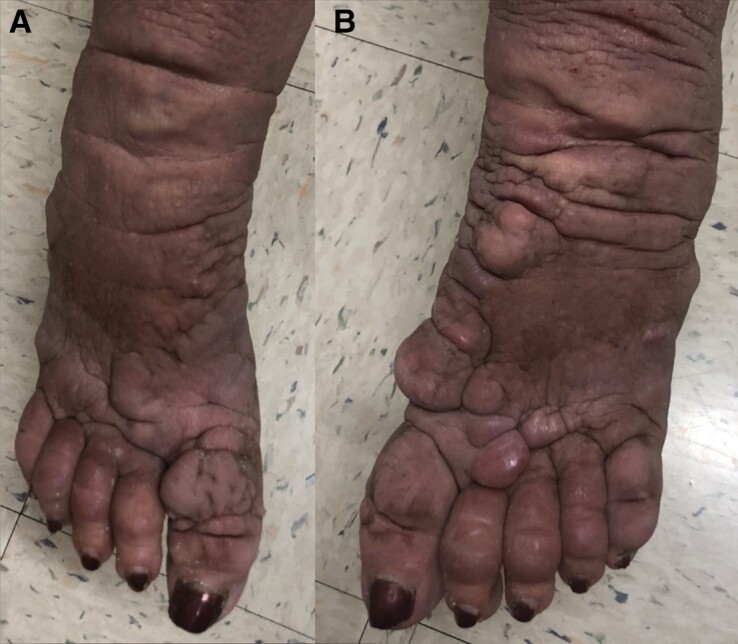
A, right foot, and B, left foot, Patient's initial photos before teprotumumab therapy. Taken in office, July 12, 2021, by Rajinderpal Chahal, MD.

The patient reported it had been several decades since she was treated with radioactive iodine ablation and has been biochemically and clinically euthyroid on 112 mcg oral levothyroxine since that time. The patient developed TED and severe myxedema of the bilateral lower extremities as a complication of her Graves disease (Fig. 1). Regarding her TED, it has been initially managed outpatient with an ophthalmologist locally, and it is noted that the initial radioactive iodine ablation did not worsen her TED. Regarding the patient's myxedema of the bilateral lower extremities, she has seen 2 dermatologists and has been seen for a specialty referral at Stanford University Medical Center. At Stanford University Medical Center, the patient was advised to take intralesional and topical steroids, although both treatments failed to resolve the lesions. The lesions substantially affected the patient's ability to wear standard shoes and thus affected her activities of daily living. Further discussion was conducted with the Cleveland Clinic that recommended the patient be referred to the National Institutes of Health (NIH) for rituximab therapy. The NIH Endocrinology division was contacted through an intermediary and was unfortunately not accepting patients at the time with severe myxedema.

## Treatment

Discussion with ophthalmology showed the patient had a clinical activity score of 4, which qualified her for teprotumumab therapy. Noted clinical manifestations of the patient's TED included bilateral exophthalmos, lid retraction, and prominent gaze. The patient was started on teprotumumab infusion therapy August 2021 for her TED. She received a total of 8 infusions, with completion February 2022. Over the course of her treatment, the patient followed up in the endocrinology office 5 times. Each time she noted improvement of her PTM. The patient noted no side effects over the course of her treatment other than mild hearing loss, which resolved after therapy was completed. Hearing loss is one of the concerning side effects of teprotumumab, and the mechanism is not currently understood, nor always reversible (5).

It is also noted that the patient has a history of non–insulin-dependent type 2 diabetes mellitus. The patient's initial glycated hemoglobin A_1c_ at the start of treatment was 6.4, and by the end of treatment was 7.2. There was no significant change or effect regarding the patient's diabetes during treatment with teprotumumab. The patient was not on medications for her diabetes, and it was controlled only with diet, exercise, and lifestyle adjustments.

**Figure 2.**
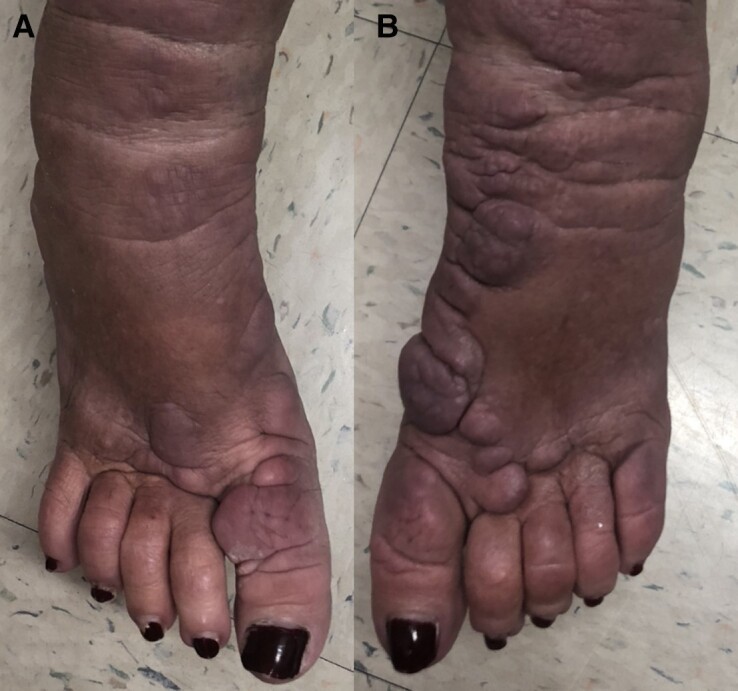
A, right foot, and B, left foot, Patient's photos during her teprotumumab course. Taken in office, January 17, 2022, by Rajinderpal Chahal, MD.

The patient noted and was quite pleased with the improvement in her bilateral lower extremity edema, nodules, and papules during the midway point of her 24-week teprotumumab infusion therapy (Fig. 2). The patient's PTM continued to improve significantly over the course of her therapy.

The patient was treated with teprotumumab, which started in early August 2021 and finished in early February 2022. She completed 8 infusions for a total of 24 weeks. Regarding the patient's postprocedural hypothyroidism, she was treated with oral levothyroxine 112 mcg daily. The patient's diabetes was treated with diet, lifestyle adjustments, and exercise. The patient was also treated with weekly 70 mg of alendronate for osteoporosis.

## Outcome and Follow-up

**Figure 3.**
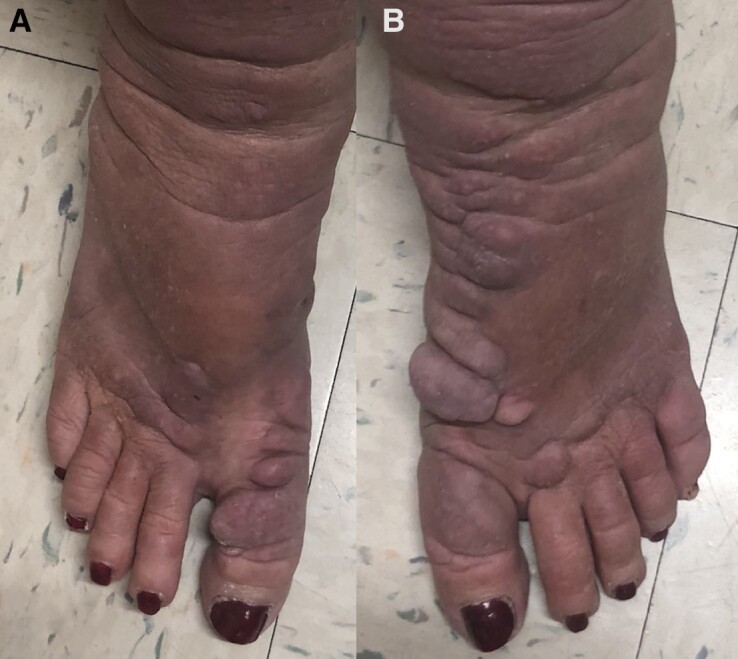
A, right foot, and B, left foot, Patient photos after completion of her teprotumumab course. Taken in office, March 17, 2022, by Rajinderpal Chahal, MD.

The patient noted considerable improvement of her PTM after completing teprotumumab infusion therapy; she reported she can now wear regular standard-fitting shoes. When comparing photos after therapy to those prior, there is significant improvement in the patient's bilateral edema, papules, and nodules (Fig. 3).

On follow-up in September 2022, the patient noted she has not noticed any worsening of her PTM since her last photo in March 2022, or regression in her quality of life. However, she stated it does feel like her extremities have slowly begun to swell again.

## Discussion

Teprotumumab is a monoclonal antibody that has been approved for use in TED, in which it has been shown to reduce proptosis. Teprotumumab's mechanism of action in TED is postulated to be due to the inhibition of IGF-R, resulting in attenuation of the fibroblast-activating antigen (6). PTM is a less common complication of thyroid disease and has not warranted the attention TED has received because of the former’s lower prevalence. The pathophysiologic manifestation of PTM is postulated to be similar to that of TED; therefore in theory, teprotumumab's mechanism of action in PTM is likely similar to that studied in TED. In this case report, we presented a patient who suffered from TED in conjunction with PTM. The patient received treatment for TED with teprotumumab infusions and concurrently noted considerable improvement in her PTM; however, she reported minimal resolution of her TED. There have been other case reports that have documented teprotumumab's role in treatment-refractory PTM. All the case reports to date studying teprotumumab in PTM have shown significant improvement in PTM (2-4). Limitations of all such case reports, including ours, has been the small number of patients being studied and lack of long-term follow-up. This is due to low PTM prevalence and due to teprotumumab being approved for use only since the year 2020. PTM prevalence in Graves' disease is 0.5% to 4.3%, and just about all cases of PTM exist in conjunction with TED (7). Future studies may guide the dosage of teprotumumab in the treatment of TED with concurrent varying severities of PTM and assess the need for treatment of disease recurrence. The total cost can also be very expensive, and something to consider when treating patients. The cost for our patient was $50 000 per infusion, which came out to $400 000 total; the total cost was noted to be completely covered by the patient's insurance for TED.

## Learning Points

- Teprotumumab is a monoclonal antibody inhibitor of IGF-1R. Currently approved for the treatment of TED, it is also a potential treatment option for patients with severe PTM.
- IGF-1R, a tyrosine kinase receptor, participates in the pathogenesis of TED, which is also a similar mechanism of action in PTM.
- Our patient, who was treated with teprotumumab for TED, showed significant resolution of PTM, which demonstrates a potential use for this specific condition.

## Data Availability

Original data generated and analyzed during this study are included in this published article.

## Abbreviations

IGF-1R: type 1 insulin-like growth factor receptor
NIH: National Institutes of Health
PTM: pretibial myxedema
TED: thyroid eye disease
